# Plasma cell‐free DNA analysis for COVID‐19 and beyond

**DOI:** 10.1002/ctd2.122

**Published:** 2022-07-26

**Authors:** Jia Ju, Kun Sun

**Affiliations:** ^1^ Institute of Cancer Research Shenzhen Bay Laboratory Shenzhen China; ^2^ Division of Life Sciences and Medicine University of Science and Technology of China Hefei China; ^3^ BGI‐Shenzhen Shenzhen China

In human, circulating cell‐free DNA (cfDNA) in peripheral blood originates from various tissues/organs. In healthy subjects, cfDNA in plasma is mostly derived from the hematopoietic system; however, in various physiological and pathological conditions, the affected tissues would release DNA into circulation, thus allowing one to utilize cfDNA as a surrogate to monitor the dynamics of the inaccessible tissues in a non‐invasive manner.^1^ Such approaches are commonly referred to as “liquid biopsies.” At present, three types of liquid biopsies are widely studied and adapted into clinical practices: non‐invasive prenatal testing for genetic disorders of the fetus by sequencing and analyzing the cfDNA in maternal plasma; detecting circulating tumour‐derived DNA (ctDNA) in the plasma of cancer patients makes it possible for early diagnosis, real‐time tumour evolution monitoring and guidance in targeted therapy; donor‐derived DNA in the plasma of transplantation recipients could be used for comprehensive monitoring of allograft rejection and injury. However, cfDNA applications in other clinical scenarios are much less explored.

The COVID‐19 pandemic lasted for more than 2 years. Due to the high variability in clinical symptoms of the COVID‐19 patients, disease severity assessment is of clinical value in providing precision healthcare and better management for the large‐number of patients. However, in most COVID‐19 patients, a handful of organs is affected simultaneously, which makes the clinical assays incompetent for accurate and rapid severity assessment. To this end, in a recent work,^2^ Bai and colleagues developed a cfDNA‐based approach for disease severity assessment of COVID‐19 patients with high translational feasibility. Bai et al. performed whole‐genome sequencing of plasma cfDNA and identified three types of features (including fragment length ratio, transcription start site coverage, and frequency of 4‐nucleotide motifs at 5’ fragment ends^3^) in 399 consecutive hospitalized COVID‐19 patients. They further combined these features with laboratory results and trained a model using machine learning approach, named *M2Model*, to distinguish critical from noncritical COVID‐19 patients with a sensitivity of 85.19% at 93.33% specificity. In addition, for clinically diagnosed critical COVID‐19 patients, Bai et al. successfully clustered them into three risk strata, and they showed that the *M2Model* was able to predict the risk of these patients towards deteriorating critical illness; moreover, survival analysis further showed that the high‐risk critical COVID‐19 patients predicted by the model required a significantly longer hospital stay than the other groups. Besides Bai et al. work, several studies from other groups had also explored the clinical applications of cfDNA as a biomarker for COVID‐19 monitoring. For instance, Cheng et al.^4^ and other groups^5^ investigated the characteristics of cfDNA in COVID‐19 patients and further developed various cfDNA‐based approaches to appraise the internal organ injuries due to COVID‐19. In particular, Cheng et al. reported that the concentration of cfDNA positively correlated with COVID‐19 disease severity and the cfDNA profile at admission allowed one to identify patients who subsequently required intensive care or died during hospitalization. Together, these studies demonstrated the clinical potential of plasma cfDNA analysis in COVID‐19, which is virtually an infectious disease.

Apart from COVID‐19, more and more studies have extended the significance of cfDNA in a broad spectrum of clinical scenarios in addition to its traditional applications (e.g., prenatal testing, cancer and organ transplantation). For examples, cfDNA analysis would provide precise subtyping of anaemia and effective monitoring of treatment responses^6^; Teo et al. showed that nucleosome signals inferred from cfDNA correlated with age‐associated changes in the epigenome in vivo ^7^ Another recent study reported that cfDNA plays an important role in the pathogenesis of systemic lupus erythematosus and showed considerable diagnostic value.^8^ In the past several years, technical advances have also been developed, which allowed one to measure more types of cfDNA molecules, such as single‐strand, ultralong and circular ones ^9^; moreover, besides concentration, genetic variation, DNA methylation profiles and copy number aberrations, the fragmentomic feature of plasma cfDNA is becoming an emerging direction in liquid biopsy, and it has been successfully implemented as novel diagnostic biomarkers for early cancer diagnosis, tumour‐of‐origin prediction, and tumour subtype.^10^ Hence, with the improvement of experimental and analytical methodologies, we believe that the next few years would witness the large expansion of the application range of plasma cfDNA in liquid biopsy, from traditional cancer fields to infectious diseases, and beyond.

## CONFLICT OF INTEREST

The authors declare that there is no conflict of interest that could be perceived as prejudicing the impartiality of the research reported.
